# Effects of High-Intensity Interval Walking on Cognitive and Physical Functions in Older Adults: A Randomized Pilot Study

**DOI:** 10.7759/cureus.68165

**Published:** 2024-08-29

**Authors:** Xinxing Li, Ji-Won Seo, Jun-Hyun Bae, Shu Jiang, Yunho Sung, Parivash Jamrasi, So Young Ahn, Sanghyuk Han, Sowoon Kim, Chaewoon Kim, In-Yeong Jang, Nur Afiqah Binti Zulkifli, Hyejung Shin, Jai Young Choi, Sang Chul Park, Wook Song

**Affiliations:** 1 Physical Education, Seoul National University, Seoul, KOR; 2 Aging, Seoul National University, Seoul, KOR; 3 Sport Science, Seoul National University, Seoul, KOR; 4 Police and Judicial Administration, Korea Senior Health Sports Association, Seoul, KOR; 5 Aging Science, Chonnam National University, Gwangju, KOR

**Keywords:** high-intensity interval walking, community-dwelling older adults, physical function performance, cognitive function scale, walking performance

## Abstract

Background: Walking is widely recognized as a prevalent form of daily exercise worldwide. However, fewer studies have explored the health outcomes of different intensities of walking exercise for older adults. Thus, the study aimed to investigate the effects of walking at different exercise intensities on body composition, emotions, cognition, and physical function among older adults.

Purpose: This study aimed to investigate the effects of walking at different exercise intensities on body composition, emotions, cognition, and physical function after eight weeks of group walking. Specifically, the study focused on the potential benefits of high-intensity interval walking (HIIW), exploring whether HIIW could have a more positive impact on the physical function and overall health of older adults compared to moderate-intensity continuous walking (MICW).

Methods: Participants aged 65 years or older were randomly assigned to either HIIW (n=13, 85% HRmax and 55% HRmax, alternating every three minutes) or MICW (n=13, 70% HRmax), engaging in group walking exercises for 30 minutes three times a week. The pre-test and post-test evaluations included body composition, cognition, emotions, and physical function.

Results: The two-minute step test showed significant improvements over time (*p*<0.0001) and time-group interaction (*p*=0.004), and sit and reach showed significant changes over time (*p*<0.0001). The independent T-test showed significant differences between the HIIW and MICW groups (two-minute step test: t (24)=1.80, *p*=0.04; sit and reach test: t (19)=3.65, *p*<0.001) at post-measurement. Additionally, no significant differences were found in body composition (weight, *p*=0.74; body mass index (BMI), *p*=0.35; body fat mass, *p*=0.45; skeletal muscle mass (SMM), *p*=0.77), emotions (geriatric depression scale (GDS), *p*=0.79; quality of life (QOL), *p*=0.54; Pittsburgh Sleep Quality Index, *p*=0.24), and cognitive function (CoSAS total score, *p*=0.25) between the HIIW and MICW groups after exercise. Grip strength, balance, 30-second chair stand, back scratch, and eight-foot up and go tests showed no significant effects in the time-group interaction.

Conclusion: Regular HIIW exercise has positive effects on physical functions such as cardiorespiratory endurance and flexibility in older adults, indicating the potential for establishing a foundation for developing customized exercise programs in the future.

## Introduction

Population aging is pervasive and enduring, especially in developed countries. South Korea is predicted to become the country with the largest proportion of the elderly population aged 65 or above in the world by 2050. Aging is well-known to be associated with a progressive decline in skeletal muscle mass (SMM), muscle function, and cardiorespiratory fitness, measured by maximum oxygen uptake (VO_2_ max), thus impairing the quality of life (QOL) [1]. Age-related frailty and muscle loss are considered to be significant public health issues in the elderly population [2]. Fortunately, before the onset of established frailty, frailty is associated with a prodromal stage known as pre-frailty, which is a dynamic and reversible condition [3]. The World Health Organization (WHO) defines healthy aging as the “process of development and maintenance of functional capacity that allows well-being at an advanced age,” emphasizing the importance of maintaining physical function and cognitive function in the elderly [4].

Physical activity is effective in delaying the decline in functional capacity due to aging, optimizing changes in body composition, improving cognitive function, and eventually improving QOL in the elderly [5]. Aerobic exercise is suggested to improve cardiovascular health, whereas resistance exercise is suggested to delay the progression of muscle loss in older adults. Flexibility exercises are also important for improving balance.

Among various exercise training methods, walking is a low-cost, low-impact exercise for the elderly. Meta-analyses focusing on elderly walking exercise demonstrated significant improvements in various aspects of body composition, including body mass index (BMI), SMM, body fat percentage, and physical function. Furthermore, cognitive function showed significant improvements through tests such as the Mini-Mental State Examination (MMSE), and emotional state through the geriatric depression scale (GDS) [6]. These findings validate the significant improvement effects of walking on body composition and physical function in the elderly.

However, excessive exercise and incorrect walking posture can increase the risk of falls and injury in the elderly. Therefore, the appropriate walking intensity needs to be studied. This study compared the effects of different walking types at different intensities using moderate-intensity and high-intensity interval walking (HIIW and MICW) on the elderly. HIIW involves performing short periods of intense walking interspersed with light-intensity rest or active recovery periods [7]. MICW can be described as fast-paced walking at moderate intensity. Typically, it involves walking with a faster and more active stride than regular walking, indicating a slightly quicker and more energetic walking style than usual [8].

Previous studies have reported significant improvements in physical performance with HIIW. For instance, a five-month HIIW program was reported to lead to a 13% increase in isometric knee extension, a 17% increase in isometric knee flexion, and a 9% increase in peak aerobic capacity for walking in older people [9]. It was also reported that ten weeks of HIIW increased cardiorespiratory fitness by 9% and reduced post-intervention disease activity in older adults with rheumatoid arthritis [10]. However, walking intensity has not been investigated separately in these studies.

Thus, the primary aim of this study is to evaluate the effects of an eight-week HIIW program on cognitive and physical function in older adults. The primary outcomes of this study include changes in body composition and physical performance, while the secondary outcomes focus on emotional and cognitive function improvements. We hypothesize that walking exercises at different intensities will lead to distinct outcomes in body composition, cognition, emotion, and physical function in older adults after the intervention period.

## Materials and methods

Study design and ethics approval

This study was designed as a randomized assessor-blind clinical trial. This study was reviewed and approved by the Institutional Review Board of Chonnam National University (IRB No. 1040198-231024-HR-156-02). The trial was conducted in accordance with the ethical principles of the Declaration of Helsinki.

**Figure 1:**
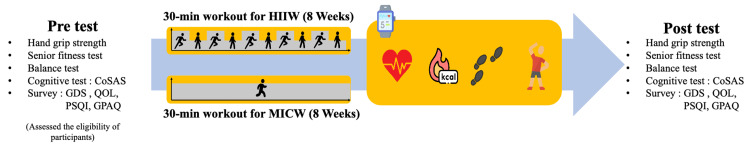
Study Design This figure indicated the experimental design of this study, comparing the effects of HIIW and MICW on older adults. The HIIW group underwent HIIW training, while the MICW group engaged in MICW. The assessments conducted included the CoSAS, GDS-K, QOL-K, PSQI-K, GPAQ, and the SFT, which comprised the chair stand test, arm curl test, two-minute step in place test, chair sit and reach test, back scratch test, and eight-foot up and go test. Hand grip strength was also measured using a dynamometer. This figure was created by Xinxing Li. HIIW, high-intensity interval walking; MICW, moderate-intensity interval walking; WHO, World Health Organization; QOL, quality of life; CoSAS, Computer Cognitive Senior Assessment System; GDS-K, the Korean version of geriatric depression scale; QOL-K, the Korean version of WHOQOL; PSQI-K, the Korean version of Pittsburgh Sleep Quality Index; GPAQ, global physical activity questionnaire; SFT, senior fitness test

Initially, the eligibility of the participants was assessed through on-site evaluations to verify compliance with the inclusion and exclusion criteria. After a one-week run-in period, those who met these criteria were randomly assigned to one of two groups: HIIW or MICW. The participants in each group were required to visit the facility three times a week to engage in walking exercises under the supervision of the researchers. To ensure the integrity of the intervention, the roles of assessors and exercise supervisors were clearly delineated to prevent any undue influence or intervention by the researchers. Following the eight-week exercise intervention period, participants were subjected to a post-test evaluation identical to the pre-test to measure any changes. The specifics of the pre- and post-test evaluations are shown in Figure 1.

Participants

We recruited individuals aged ≥ 65 years who provided written consent to participate in our study. However, we excluded those with 1) BMI <18.5 or >30 kg/m^2^; 2) recent high-intensity exercise; 3) lower extremity musculoskeletal issues; 4) inability to converse or walk due to dementia or Parkinson’s disease; 5) surgery within the last six months; 6) severe cerebrovascular, cardiac, or pulmonary diseases; 7) conditions such as hyperlipidemia, asthma, diabetes, anemia, thyroid, cardiovascular, renal, liver, musculoskeletal, or neurological disorders, or those requiring anticoagulants; 8) difficulty using a smartphone; and 9) any other reason researchers deemed them unsuitable for the study. Considering a dropout rate of 30%, we recruited 26 subjects who met the inclusion criteria.

Interventions 

All participants underwent a pre-test and then engaged in eight weeks of weekly HIIW or MICW. The real-time heart rate for setting exercise intensity during the intervention was measured using a smartwatch Mi Band 7 (Mi Band 7, Xiaomi, Beijing, China). The maximum heart rate (HRmax) of each participant (220 years old) was calculated individually. The HIIW group completed a 30-minute session that alternated between three minutes of HIIW at 85% of HRmax and three minutes of low-intensity walking at 55% of HRmax, completing five intervals in total. The MICW was instructed to walk for 30 minutes at 70% of HRmax. Both groups performed a five-minute warm-up and cool-down at 45% HRmax. If the participant's heart rate exceeded the target, the walking speed was adjusted to modulate the intensity.

To ensure that participants maintained proper form during the exercise, we stationed two supervisors at 200-meter intervals along the 400-meter track. Each supervisor monitored the participants’ smartwatches to check their heart rates as they passed by. A third supervisor moved around the track, keeping track of time and providing real-time feedback to the participants.

Additionally, to maximize adherence to the protocol, participants were instructed to check their heart rates every time they passed a supervisor. This systematic approach allowed us to ensure that participants maintained the correct exercise intensity and followed the protocol consistently throughout the intervention.

Measurements

Anthropometric Measures

Body weight, SMM, BMI, and body fat mass were measured using a bioelectrical impedance analyzer called InBody770 (InBody770, InBody, Seoul, Republic of Korea). An automatic stadiometer (BSM370, InBody, Seoul, Republic of Korea) was used to assess the participant's height. Blood pressure (BPBIO320, InBody, Seoul, Republic of Korea) and body temperature were recorded after a 10-minute rest upon arrival at the laboratory. All measurements were consistent with the same researcher at the same time point in each visit, and the same machine was used to measure the variables.

Physical function

Physical function was assessed using the senior fitness test (SFT), muscle strength, and balance. The SFT, which had been introduced by Rikli and Jones, was conducted to evaluate the fitness level of older people [11]. This test assesses key components of functional fitness, including strength, flexibility, agility, and balance, which are vital for preserving independence and QOL in older individuals. It consists of functional movement tasks such as the chair stand test, arm curl test, two-minute step in place test, chair sit and reach test, back scratch test, and eight-foot up and go test.

Muscle strength was measured using hand grip strength. Participants were instructed to grab the digital hand grip dynamometer (TKK-5401, Takei, Tokyo, Japan), straighten their arms, and pull the handle hard while maintaining the arms at 15° from the torso. They posed for 5 s, as instructed, with maximum force. The average record was calculated after completing two trials on each side [12].

A balance test was conducted using MFT challenge disc 2.0 (MFT Bodyteamwork, Vienna, Austria) equipment. Participants were required to stand still for 20 s on the disc, and the balance score was automatically calculated [13].

Psychological health and physical activity

Depression, QOL, sleep quality, and physical activity levels were self-reported using questionnaires. Depression was evaluated using the Korean version of the geriatric depression scale (GDS-K) to determine the degree of depression among participants [14]. QOL was measured using the abbreviated the Korean version of the WHOQOL, which encompasses the overall life and physical, psychological, social, and environmental domains [15]. Sleep quality over the past month was assessed using the Korean version of the Pittsburgh Sleep Quality Index (PSQI-K) [16]. Additionally, participants' physical activity levels were evaluated using the global physical activity questionnaire (GPAQ) [17].

Cognitive function

Cognitive function was evaluated using the Computer Cognitive Senior Assessment System (CoSAS, RPiO, Seoul, Republic of Korea). CoSAS is a simple test used as a mobile platform to detect cognitive dysfunction [18]. It consists of 55 questions in six areas: orientation, memory, attention, visual perception, language ability, and high-level ability, with a total score of 64.

Statistical analyses

Statistical analysis was performed using Prism 9 (v.10.2.0, GraphPad, MA, USA) software and SPSS (v.29, IBM, IL, USA) for Windows. A 2×2 repeated measures analysis of variance (ANOVA) was performed to determine the effects of HIIW compared to MICW within the time factor. For simple two-way interactions and main effects, Bonferroni adjustment was used, and statistical significance was set at *p*<0.05. After analyzing the homogeneity of the measurements (*p*>0.05) between HIIW and MICW, all data are presented as the mean ± standard deviation (SD). All data were normally distributed (*p*>0.05) using the Kolmogorov-Smirnov (K-S) normality test. In addition, the normality of sex distribution was assessed using Fisher’s exact test (*p*>0.05). Additionally, Levene’s test (standard of mean) for equality of variances was used to assess the variance homogeneity. Before exercise training, significant measurements were assessed to identify variances using the Welch method after Levene’s test for the equality of variances. Statistical significance was set at *p*<0.05.

## Results

The results of basic characteristics between HIIW and MICT groups

As shown in Table 1, all measurements (n=26 (100%)) were not significantly different between HIIW (n=13 (50%)) and MICW (n=13 (50%)), except for height measurement (K-S=0.23, df=13, *p*=0.02) in HIIW before exercise training. However, this study used a parametric statistical method because height was not a critical measurement and BMI, which was related to height measurement, was not significantly different (K-S=0.11, df=13, *p*=0.20). All measurements in the Leve test showed that BMI (Levene=6.403, F(1, 24)=0.02) and body fat mass (Levene=7.26, dF(1, 24)=0.01) were significantly different. The statistical Welch method indicated both BMI (Welch=4.18, dF(1, 19.68)=0.05) and body fat mass (Welch=2.34, dF(1, 19.48)=0.14) was not significantly different before exercise training. Thus, all measurements showed normal distribution and equality of variance.

**Table 1:** Basic Characteristics of Subjects Between HIIW and MICW Groups ^a^HIIW group ^b^MICW group *p<0.05 BMI, body mass index; SBP, systolic blood pressure; BFM, body fat mass; DBP, diastolic blood pressure; SMM, skeletal muscle mass; K-S, Kolmogorov-Smirnov; MICW, moderate-intensity continuous walking; HIIW, high-intensity interval walking

Measurement	HIIW group (n=13) (Mean SD)	MICT group (n=13) (Mean±SD)	K-S^a^	K-S^b^	*P*-value^a^	*P*-value^b^
Age	74±4.3	74.8±5.1	0.18	0.18	0.20	0.20
Height (cm)	158.4±6.2	161.1±8.3	0.10	0.26	0.20	0.02*
Weight (kg)	62.8±9.1	59.6±6.9	0.19	0.12	0.22	0.20
BMI (kg/m^2^)	25.2±3.4	23.0±2.0	0.16	0.11	0.20	0.20
BFM (kg)	21.6±7.1	18.2±4.2	0.17	0.12	0.20	0.20
SMM (kg)	21.8±3.2	22.1±4.2	0.15	0.18	0.20	0.20
SBP (mmHg)	136.7±12.8	131.8±12.8	0.23	0.17	0.05	0.20
DBP (mmHg)	72.3±9.9	69.1±7.5	0.11	0.19	0.20	0.20
GPAQ	1087.7±132.1	867.7±139.0	0.17	0.20	0.20	0.16

Results of body composition between HIIW and MICW groups after exercise training

**Figure 2:**
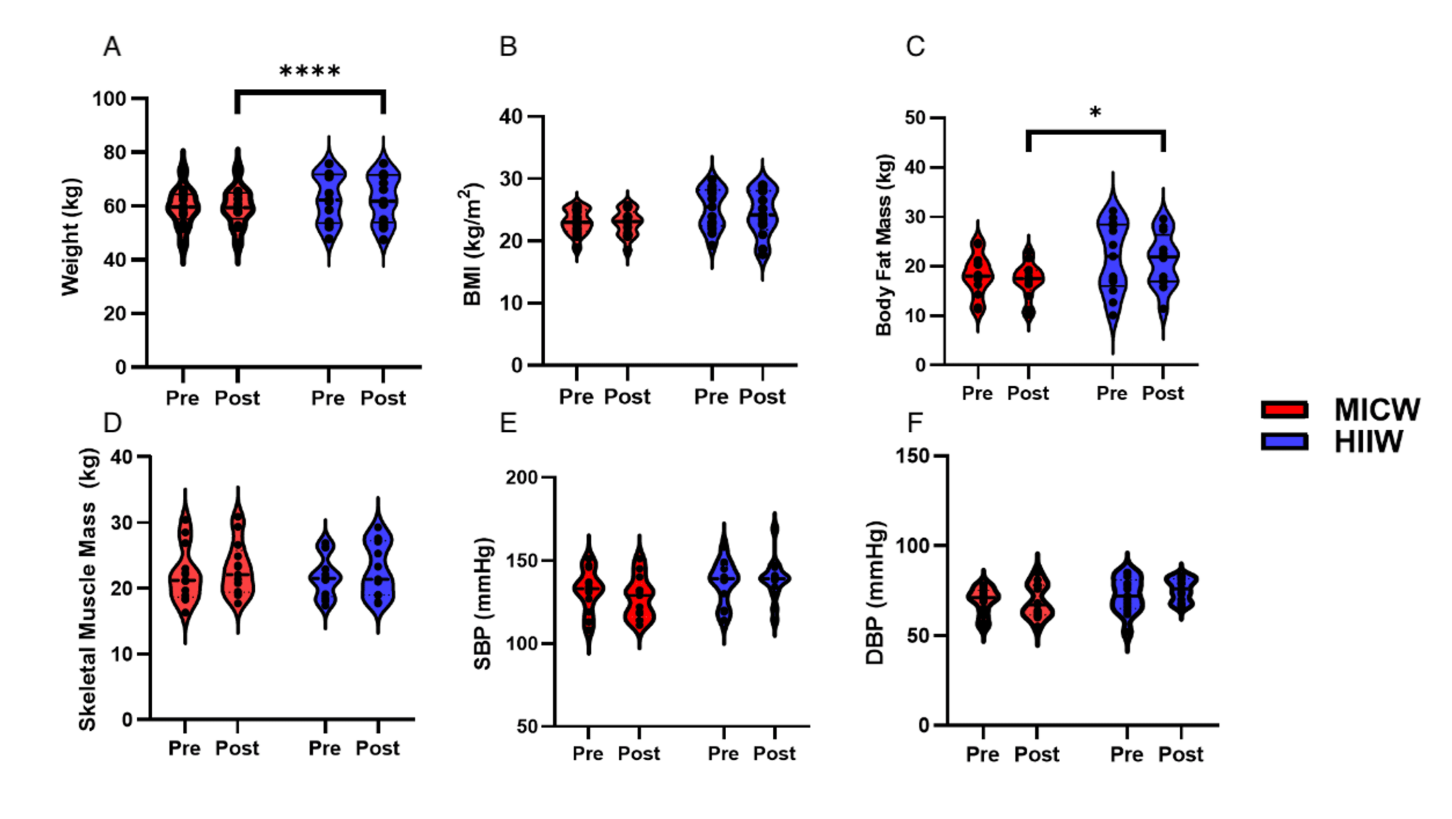
The Results of Body Composition and Blood Pressure Between MICW and HIIW Groups This figure indicated the body composition and blood pressure between the pre- and post-time within MICW and HIIW groups. A) Weight (kg) difference between the pre- and post-time within MICW and HIIW groups. B) The different BMI (kg/m^2^) between the pre- and post-time within MICW and HIIW groups. C) The different results of body fat mass (kg) between the pre- and post-time within MICW and HIIW groups. D) The results of different SMM (kg) between the pre- and post-time within MICW and HIIW groups. E) Different SBP (mmHg) between the pre- and post-time within MICW and HIIW groups. F) The results of different DBP (mmHg) between the pre- and post-time within MICW and HIIW groups. All statistical values were represented. *p<0.05 **p<0.01 ***p<0.001 MICW, moderate-intensity continuous walking; HIIW, high-intensity interval walking; BMI, body mass index; SBP, systolic blood pressure; DBP, diastolic blood pressure; SMM, skeletal muscle mass

Weight (Figure 2A) was not significantly different in the interaction (F(1, 24)=0.12, *p*=0.74), time (F(1, 24)=0.07, *p*=0.80), and group (F(1, 24)=1.02, *p*=0.32) between the HIIW and MICW groups after exercise training. BMI (Figure 2B) did not significantly differ in the interaction (F(1,24)=0.93, *p*=0.35), time (F(1,24)=1.54, *p*=0.23), and group (F(1, 24)=2.98, *p*=0.10) between the HIIW and MICW groups after exercise training. Body fat mass (kg) (Figure 2C) was not significantly different in the interaction (F(1, 23)=0.60, *p*=0.45), time (F(1, 23)=0.73, *p*=0.40), and group (F(1, 24)=4.04, *p*=0.06) between HIIW and MICW after exercise training. SMM (Figure 2D) showed a significant change over time (F(1, 24)=5.97, *p*=0.02), with no significant interaction between time and group (F(1, 24)=0.09, *p*=0.77) or group effect (F(1, 24)=0.02, *p*=0.89). Systolic blood pressure (SBP) (Figure 2E) showed no significant changes over time (F(1, 24)=0.48, *p*=0.50), no significant group effect (F(1, 24)=2.83, *p*=0.11), and no significant time-group interaction (F(1, 24)=2.12, *p*=0.16). Diastolic blood pressure (DBP) (Figure 2F) analysis showed no significant changes over time (F(1, 24)=1.39, *p*=0.25), no significant group effect (F(1, 24)=2.55, *p*=0.12), and no significant time-group interaction (F(1, 24)=1.39, *p*=0.25). The exercise training study found no significant differences in weight, BMI, body fat mass, SBP, DBP, or resting pulse rate (RPR) between the HIIW and MICW groups, except for a significant increase in SMM over time (Figure 2).

Results of GDS-K, OQL-K, and PSQI-K between HIIW and MICW groups after exercise training

**Figure 3:**
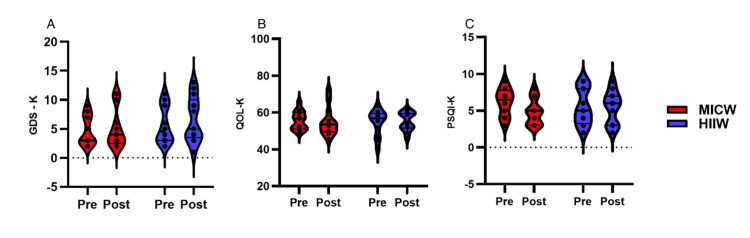
The Results of GDS-K, QOL-K, and PSQI-K Between MICW and HIIW Groups This figure showed the GDS-K, QOL-K, and PSQI-K scores between the pre- and post-time within MICW and HIIW groups. A) The results of different GDS-K scores between the pre- and post-time within MICW and HIIW groups. B) The results of different QOL-K between the pre- and post-time within MICW and HIIW groups. C) The results of different PSQI-K scores between the pre- and post-time within MICW and HIIW groups. All statistical values were represented. *p<0.05 **p<0.01 ***p<0.001 GDS-K, the Korean version of the geriatric depression scale; QOL-K, the Korean version of WHO quality of life, PSQI-K, the Korean version of the Pittsburgh Sleep Quality Index; WHO, World Health Organization; MICW, moderate-intensity continuous walking; HIIW, high-intensity interval walking

The Korean version of the geriatric depression scale (GDS-K) (Figure 3A) demonstrated no statistically significant effects on time (F(1, 12)=0.42, *p*=0.53), group (F(1, 12)=2.21, *p*=0.16), or the interaction between time and group (F(1, 9)=0.07, *p*=0.79). The Korean version of WHOQOL (QOL-K) (score) (Figure 3B) was not significantly different between the groups (F(1, 24)=0.07, *p*=0.80), group (F(1, 24)=0.03, *p*=0.87), and time-group interaction (F(1, 24)=0.40, *p*=0.54). The PSQI-K (Figure 3C) was also no significant differences in time (F(1, 24)=0.29, *p*=0.60), group (F(1, 24=0.03, *p*=0.86), or time-group interaction (F(1, 24)=1.47, *p*=0.24) (Figure 3).

Results of physical function between HIIW and MICW Groups after exercise training

**Figure 4:**
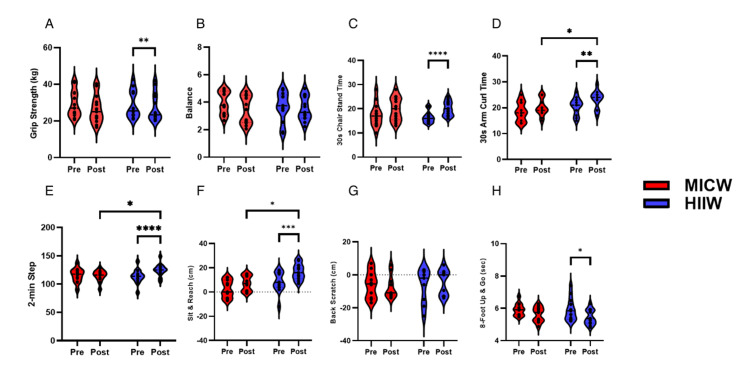
The Results of Physical Functions Between MICW and HIIW Groups This figure indicated the different physical function results between pre- and post-time within MICW and HIIW groups. A) The results of different grip strength (kg) between the pre- and post-time within MICW and HIIW groups. B) The results of different balance (score) between the pre- and post-time within MICW and HIIW groups. C) The results of different 30 s chair stand times (sec) between the pre- and post-time within MICW and HIIW groups. D) The results of different 30s arm curl times (sec) between the pre- and post-time within MICW and HIIW groups. E) The results of different two-min steps (count) between the pre- and post-time within MICW and HIIW groups. F) The results of different sit and reach (cm) between the pre- and post-time within MICW and HIIW groups. G) The results of different back stretch (cm) between the pre- and post-time within MICW and HIIW groups. H) The results of different eight-foot up and go (sec) between the pre- and post-time within MICW and HIIW groups. All statistical values were represented *p<0.05 **p<0.01 ***p<0.001 MICW, moderate-intensity continuous walking; HIIW, high-intensity interval walking

The results of physical function between HIIW and MICW groups after exercise training are shown in Figure 4. The analysis showed a significant decrease in grip strength (Figure 4A) over time (F(1, 24)=41.24, *p*<0.001), with no significant time-group interaction (F(1, 24)=2.92, *p*=0.10) and differences between the HIIW and MICW groups (F(1, 24)=0.02, *p*=0.89). Balance (Figure 4B) measurements revealed a significant improvement over time (F(1, 24)=4.91, *p*=0.04), with no significant differences between the HIIW and MICW groups. In addition to the significant improvement in 30-second chair stand test times (Figure 4C) over time (F(1, 23)=59.73, *p* < 0.0001) without group differences (F(1, 24)=0.10, *p*=0.76) and interaction effect (F(1, 23)=1.88, *p*=0.18). 

30-second arm curl test (count) (Figure 4D) revealed significant differences over time (F (1, 23)=11.20, *p*=0.00) and between groups (F(1, 23)=4.72, *p*=0.04), without a significant time-group interaction (F(1, 23)=0.56, *p*=0.46). The post-measurements were not significantly different between the HIIW and MICW groups (t (24)=1.54, *p*=0.07, mean difference=2.31, mean difference of 95% CI -0.79-5.40).

The two-minute step test (Figure 4E) showed significant improvements over time (F(1, 24)=23.14, *p*<0.0001) and time-group interaction (F(1, 24)=10.28, *p*=0.00) except for no significant difference between the groups (F(1, 24)=0.41, *p*=0.53). The post-measurements were significantly different between the HIIW and MICW groups (t(24)=1.80, *p*=0.04, mean difference=7.15, mean difference of 95% CI -1.05-15.36).

In the sit and reach test (cm) (Figure 4F), significant changes over time were identified (F(1, 19)=24.54, *p*<0.0001), alongside notable group differences (F(1, 19)=11.58, *p*=0.00), but no significant time-group interaction was found (F(1, 19)=1.40, *p*=0.25). The post-measurements were significantly different between the HIIW and MICW groups (t(19)=3.65, *p*=<0.001, mean difference=10.23, mean difference of 95% CI 4.37-16.09). 

The back-scratch (Figure 4G) was not significantly affected by time (F(1, 17)=0.13, *p*=0.72), group (F(1, 17)=0.11, *p*=0.75), or time-group interaction (F(1, 17)=2.88, *p*=0.11). 

The eight-foot up and go test (Figure 4H) showed a significant effect of time (F(1, 22)=8.35, *p*=0.01), indicating an improvement in function over time. However, no significant effects were found for the group (F(1, 22)=0.33, *p*=0.57) or time-group interaction (F(1, 22)=0.33, *p*=0.57). The HIIW group showed improvements only in the two-minute step test and the sit and reach (cm) measurements.

Results of CoSAS between HIIW and MICW Groups after exercise training

**Figure 5:**
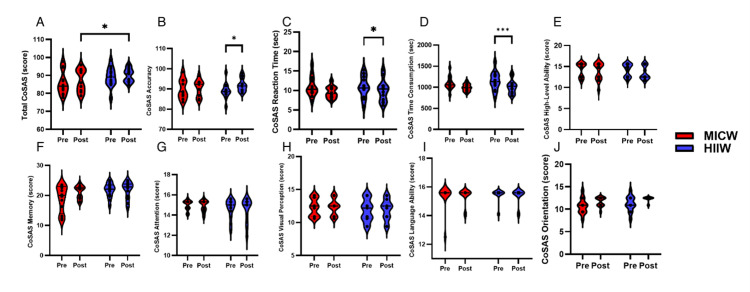
The Result of CoSAS Cognitive Function Between MICW and HIIW Groups This figure indicated the different CoSAS cognitive function results between pre- and post-time within MICW and HIIW groups. A) The results of different total CoSAS (score) between the pre- and post-time within MICW and HIIW groups. B) The results of different CoSAS accuracy (score) between the pre- and post-time within MICW and HIIW groups. C) The different CoSAS reaction time (sec) results between the pre- and post-time within MICW and HIIW groups. D) The results of different CoSAS consumption times (sec) between the pre- and post-time within MICW and HIIW groups. E) The different CoSAS high-level ability (score) results between the pre- and post-time within MICW and HIIW groups. F) The different results of CoSAS memory (score) between the pre- and post-time within MICW and HIIW groups. G) The different results of CoSAS attention (score) between the pre- and post-time within MICW and HIIW groups. H) The different results of CoSAS visual perception (score) between the pre- and post-time within MICW and HIIW groups. I) The results of different CoSAS language ability (score) between the pre- and post-time within MICW and HIIW groups. J) The different results of CoSAS orientation (score) between the pre- and post-time within MICW and HIIW groups. All statistical values were represented. *p<0.05 **p<0.01 ***p<0.001 CoSAS, Computer Cognitive Senior Assessment System; MICW, moderate-intensity continuous walking; HIIW, high-intensity interval walking

The CoSAS total score (Figure 5A) showed no significant changes over time (F(1, 24)=2.37, *p*=0.14), group effect (F(1, 24)=1.07, *p*=0.31), or time-group interaction (F(1, 24)=1.39, *p*=0.25). The CoSAS accuracy (Figure 5B) indicated that there was no significant difference over time (F(1, 24)=3.62, *p*=0.07), group effect (F(1, 24)=0.15, *p*=0.70), and time-group interaction (F(1, 24)=0.22, *p*=0.65). The CoSAS reaction time (Figure 5C) was not significantly different between the groups (F(1, 24)=2.59, *p*=0.12), group (F(1, 24)=0.50, *p*=0.49), and time-group interaction (F(1, 24)=0.00, *p*=0.99). CoSAS consumption time (Figure 5D) was not significantly different over time (F(1, 24)=2.65, *p*=0.12), group effect (F(1, 24)=0.00, *p*=0.97), or time-group interaction (F(1, 24)=1.09, *p*=0.31). The CoSAS orientation (Figure 5J) showed a significant difference in time (F(1, 24)=6.30, *p*=0.02), except for the time-group interaction (F(1, 24)=0.03, *p*=0.87) and group effect (F(1, 24)=2.79, *p*=0.11). CoSAS memory (Figure 5F) did not significantly differ over time (F(1, 24)=1.36, *p*=0.26) or time-group interaction (F(1, 24)=1.32, *p*=0.26), except in the group effect (F(1,24)=4.86, *p*=0.04). CoSAS attention (Figure 5G) was not significantly different over time (F(1, 24)=0.20, *p*=0.66), group effect (F(1, 24)=0.13, *p*=0.72), and time-group interaction (F(1, 24)=0.55, *p*=0.46). CoSAS visual perception (Figure 5H) was not significant over time (F(1, 24)=0.07, *p*=0.79), group effect (F(1, 24)=0.85, *p*=0.37), or time-group interaction (F(1, 24)=0.00, *p*=0.95). CoSAS language ability (Figure 5I) showed no significant differences in time (F(1, 24)=0.09, *p*=0.77), group effect (F(1, 24)=0.014, *p*=0.71), or time-group interaction (F(1, 24)=0.11, *p*=0.74). CoSAS higher-level ability (Figure 5E) did not differ significantly over time (F(1, 24)=1.63, *p*=0.21), group effect (F(1, 24)=0.15, *p*=0.70), and time-group interaction (F(1, 24=0.04, *p*=0.84). In a study evaluating cognitive skills through the CoSAS, only orientation showed a significant improvement over time, with no significant changes observed in the total score, accuracy, reaction time, consumption time, memory, attention, visual perception, language ability, and higher-level ability, except for a significant group effect in memory (Figure 5).

## Discussion

This study evaluated the effects of a walking exercise program of varying intensities on physical function, body composition, cognition, and emotion in older adults. The findings offer insights into the potential benefits of different intensity walking exercises for this population.

We found that both types of walking exercise significantly improved the cardiorespiratory fitness of our subjects over time. However, HIIW showed a superior positive impact at the end of the intervention. Similar to our finding, Bartlett and David et al. reported that 10 weeks of HIIW training in older adults resulted in a 9±4% increase in both relative and absolute cardiorespiratory fitness [10]. On the other hand, it has been reported that there is a sex difference in the improvement of aerobic fitness. They found that, compared to control subjects, five months of HIIW significantly improved VO_2_ max in men but not in women [19].

As expected, since the training protocol focused primarily on walking and did not target the upper body muscles extensively, grip strength, back stretch, and the 30-second arm curl test showed no significant differences between the groups. In contrast to our findings, a study demonstrated improvements in grip strength, sit-to-stand performance, and arm curl after 12 weeks of normal walking in elderly women [20].

In terms of lower limb function, significant improvements over time were observed in all the 30-second chair stands, eight-foot up and go tests, and balance tests for all participants. In addition, conducting the intervention in a well-controlled environment, such as a 400-meter track with consistent supervision, ensured uniform application of the exercise protocols. This careful control helped to minimize variability and strengthen the validity of the findings. However, it appears that the intensity of our protocols did not strongly differentiate between the groups in terms of these improvements. However, Bartlett et al. reported that in older adults with rheumatoid arthritis, 10 weeks of HIIW training resulted in a reduction in the time needed to walk 400 m and an increase in the number of chair stands completed within 30 s [10]. Although we did not observe an improvement in balance in either group, Figueiredo et al. reported changes in the Berg Balance Scale after four weeks of walking [20].

Remarkably, the HIIW group showed greater improvement in the sit and reach test, which measures the extensibility of the hamstrings and lower back. Regular walking can prevent hamstring tightness. Previous research has indicated that backward walking increases hamstring flexibility and expands the lower back's range of motion in both young individuals and adults with knee osteoarthritis aged 40 to 70 years [21].

Regarding body composition, no significant differences were observed between the HIIW and MICW groups after exercise training. There were no notable disparities in weight, BMI, body fat mass, SMM, blood pressure, or RPR between the two groups [22].

However, it is worth noting that there was a significant increase in SMM over time in the HIIW and MICW groups. A previous study compared protein synthesis rates after a single session of high-intensity interval training (HIIT), resistance exercise, or MICT in untrained older men. They found that myofibrillar protein synthesis significantly increased 24 and 48 h after HIIT compared with that at rest [23]. While the increase was lower with resistance exercise (50% vs. 80%), it was higher than with MICT (10%) [23]. Furthermore, only HIIT led to increased sarcoplasmic protein synthesis at 24 h post-exercise (25%), possibly due to increased mitochondrial protein synthesis. This suggests that skeletal muscle remodeling with HIIT may go beyond the established changes in oxidative capacity and substrate metabolism [24]. An eight-week walking exercise program has been reported to result in a mean increase of 2.4% in SMM, a key muscle component [25]. This finding underscores the effectiveness of walking exercises for enhancing muscle health. However, unlike the improvement in walking speed, there was no significant association with changes in body fat. This suggests that, while the enhancement in walking speed is primarily associated with increases in muscle mass and strength, it is not correlated with changes in body fat [26]. This interpretation underscores the practical significance of the findings, offering crucial insights for customizing exercise programs and improving strategies for managing the health of the elderly. Additionally, these outcomes have the potential to shape societal policies and individual health decisions aimed at promoting healthier aging.

In this study, questionnaires were used to measure the QOL, sleep quality, and depression levels of elderly Korean individuals using the Korean versions of the WHOQOL, PSQI, and GDS, which have demonstrated validity and reliability. Our study showed non-significant improvements in QOL after eight weeks of HIIW and MICW training. In contrast, another study demonstrated that walking exercise significantly enhanced the QOL for older adults, despite the study’s duration of four months and one-hour sessions, which considerably exceeded the duration and intensity of our intervention [27].

Thirty minutes of HIIW and MICW exercises in our study did not show a significant difference after eight weeks of exercise intervention. This result contrasts with the findings of a previous study, which indicated that 1 h of walking exercise improved the depression scale for older adults in rural areas [28]. The variation in the exercise duration between the two studies may account for this difference.

Our study found no significant differences in sleep quality among older adults from HIIW and MICW training. However, Bullock et al. reported significant improvement in the MICW group for enhancing sleep quality, while the HIIW group showed no improvement [29]. The variations could be due to 70% of the sample being reported as poor sleepers.

The results suggested that walking exercise did not significantly improve cognitive function. This may be due to participants already possessing good cognitive function at baseline, limiting the intervention's observable impact [30]. Mielke et al. proposed that variations in study findings could be partly due to differences in participant demographics, including individuals with varying cognitive statuses from normal cognition to mild cognitive impairment or Alzheimer's disease [31].

Although this study was conducted with a relatively small sample size and over a limited duration, it serves as a crucial pilot study that lays the groundwork for larger-scale interventions. The data gathered here provide a foundation for refining future research designs and exploring more targeted exercise programs for the elderly.

## Conclusions

In conclusion, our study found that HIIW significantly improves physical function, particularly cardiorespiratory fitness and flexibility, more than MICW. These findings suggest that HIIW is a more effective method for enhancing physical health in older adults. Additionally, while both HIIW and MICW positively impacted skeletal muscle mass, no significant differences were observed in weight, BMI, body fat mass, blood pressure, or resting pulse rate between the two groups. Importantly, neither walking program significantly influenced cognitive function, QOL, sleep quality, or depression, indicating that these areas may require longer intervention periods or additional supportive measures to observe notable changes.

The definitive outcome of our findings is that HIIW can be more effective in improving specific physical health parameters in older adults. This has practical implications for developing customized exercise programs tailored to this population. Future research should further explore the potential of HIIW interventions, potentially combining them with cognitive and psychological training to achieve more comprehensive health benefits. These insights are crucial for shaping exercise recommendations and policies aimed at promoting healthier aging in the elderly population.
